# Dynamic respiratory muscle function in late-onset Pompe disease

**DOI:** 10.1038/s41598-019-54314-8

**Published:** 2019-12-12

**Authors:** Barbara K. Smith, Shannon Allen, Samantha Mays, A. Daniel Martin, Barry J. Byrne

**Affiliations:** 1 Department of Physical Therapy, University of Florida, Gainesville, Florida, USA; 2 Department of Pediatrics, University of Florida, Gainesville, Florida, USA; 3 Departments of Health Science and Physician Assistant Studies, University of Florida, Gainesville, Florida, USA

**Keywords:** Neuromuscular disease, Respiratory signs and symptoms

## Abstract

Maximal inspiratory pressure (PI_MAX_) reflects inspiratory weakness in late-onset Pompe disease (LOPD). However, static pressure tests may not reveal specific respiratory muscle adaptations to disruptions in breathing. We hypothesized that dynamic respiratory muscle functional tests reflect distinct ventilatory compensations in LOPD. We evaluated LOPD (n = 7) and healthy controls (CON, n = 7) during pulmonary function tests, inspiratory endurance testing, dynamic kinematic MRI of the thorax, and ventilatory adjustments to single-breath inspiratory loads (inspiratory load compensation, ILC). We observed significantly lower static and dynamic respiratory function in LOPD. PI_MAX_, spirometry, endurance time, and maximal diaphragm descent were significantly correlated. During single-breath inspiratory loads, inspiratory time and airflow acceleration increased to preserve volume, and in LOPD, the response magnitudes correlated to maximal chest wall kinematics. The results indicate that changes in diaphragmatic motor function and strength among LOPD subjects could be detected through dynamic respiratory testing. We concluded that neuromuscular function significantly influenced breathing endurance, timing and loading compensations.

## Introduction

Pompe disease is a hereditary lysosomal storage disorder prompted by a mutation of the gene that encodes acid α-glucosidase (GAA), an enzyme essential for the degradation of lysosomal glycogen^1^. A shortage or absence of GAA leads to glycogen accumulation and ultimately impairment of the contractile units present in skeletal, smooth, and cardiac muscle cells^2^. In the late-onset Pompe phenotype (LOPD), respiratory muscle dysfunction progresses over years to decades and leads to sleep disordered breathing, ineffective airway clearance, and progressive ventilatory insufficiency^3,4^. Preferential phrenic paresis occurs early in Pompe^5^ and is closely followed by abdominal muscle dysfunction^6,7^. As in most neuromuscular diseases, respiratory insufficiency is the primary contributor to morbidity and mortality^8,9^.

Despite the high prevalence of respiratory problems in patients with LOPD, there are limited methods to comprehensively and objectively evaluate changes in respiratory muscle function. Maximal inspiratory pressure (PI_MAX_) is the clinical estimate of inspiratory muscle strength. The PI_MAX_ maneuver is noninvasive and does not require bulky or expensive equipment, which makes it useful for patient care and multi-site clinical trials. However, PI_MAX_ is influenced by learning as well as the starting lung volume, making it prone to measurement error^10^. Moreover, the static PI_MAX_ maneuver does not reflect the dynamic properties of diaphragm and respiratory accessory muscle contractions or take into account the effects of airway and pulmonary mechanics. In ventilator-dependent patients with infantile Pompe, we found that neither initial PI_MAX_ nor changes in PI_MAX_ corresponded with a reduced requirement for mechanical ventilation or airway clearance assistance^11,12^.

In contrast to a static measure of respiratory muscle strength, dynamic tests incorporate changes in respiratory pressure or volume over a finite period such as a tidal breath. Dynamic function of the respiratory muscles can be evaluated through different approaches. First, the respiratory timing, flow, and volume are transiently altered during brief respiratory mechanical loads. These modifications in the breathing pattern, termed inspiratory load compensation (ILC), depend upon the nature and magnitude of the imposed load. During ILC and respiratory muscle fatigue tests, pressure threshold loads deliver a flow-independent pressure load^13^. The initial phase of a threshold-loaded inspiratory effort is quasi-isometric, and becomes dynamic when sufficient pressure has been generated to open the inspiratory valve^14,15^. ILC resembles the requirements of the respiratory muscles when challenged by a finite load, such as a partial obstruction of the airway by mucus. ILC tests may offer insight into compensatory changes in the breathing pattern with changes in function such as disease progression or in response to exercise training^16^.

A second method to evaluate the dynamic function of the respiratory pump is with magnetic resonance imaging (MRI). Static MRI images of the thorax at total lung capacity and residual volume indicate that lung volumes and diaphragm descent are reduced in LOPD, and these appear to correlate with clinical pulmonary function tests such as forced vital capacity^3,5,7^. Dynamic mode (cinematic) thoracic MRI has also been employed to quantify rate of change of diaphragm descent and disease severity in pulmonary disease, scoliosis, and Duchenne muscular dystrophy^17–20^. Dynamic scans are advantageous because they do not require static breath-holds at fixed volumes to calculate respiratory muscle excursions, and multiple repeated breaths can be acquired and averaged over a short duration. Moreover, dynamic imaging can discern changes in the configuration of the chest related to diaphragm descent versus expansion of the chest wall.

This study compared respiratory function in LOPD to that of unaffected, age- and gender-matched controls, using ILC and dynamic MRI testing. The primary purpose of this project was to determine the relationships between pulmonary function tests, ILC responses to mechanical loads, and dynamic-mode MRI as assessments of dynamic function of the respiratory muscles in adult Pompe disease. We hypothesized that ILC volume responses and endurance will correlate both with PI_MAX_ and with MRI measurements of diaphragm descent. Further, we hypothesized that dynamic respiratory muscle function in LOPD would differ significantly from unaffected controls.

## Results

### Demographic characteristics and pulmonary function

The demographic characteristics of the sample are listed on Table 1. In patients, the predicted FVC (LOPD: 59 ± 22%, CON: 110 ± 45%, p < 0.005), FEV_1_ (LOPD: 60 ± 20%, CON: 108 ± 41%, p < 0.001), PI_MAX_ (LOPD: 59 ± 20 cm H_2_O, CON: 95 ± 45 cm H_2_O, p < 0.05), and PE_MAX_ (LOPD: 77 ± 31 cm H_2_O, CON: 111 ± 41 cm H_2_O, p < 0.05) were significantly below the control subjects. LOPD subjects averaged 5.7 (±4.4) years from diagnosis at testing, 5 were treated with enzyme replacement therapy (ERT), and 4 required nighttime ventilatory assistance (CPAP = 1, BiPAP = 3). Body mass index, ETCO_2_, and minute ventilation did not differ between groups.

**Table 1:** Demographic Characteristics of the sample.

	Age (years)	Gender	BMI (kg/cm^2^)	FVC pred (%)	FEV_1_ Pred (%)	PI_MAX_ (cmH_2_O)	PE_MAX_ (cmH_2_O)	ETCO_2_ (mm Hg)	V_E_ (L/min)	Age at Diagnosis (years)	Use of ERT	Nighttime Support?
	**Pompe Subjects**											
P1	50	F	27.12	69.7	69	49	62	34	9.7	42	yes	NO
P2	47	F	25.4	67	66.2	69	79	45	9.9	37	yes	CPAP
P3	52	M	26.4	36.7	35.9	36	88	48	10.2	40	no	BiPAP
P4	32	M	26.58	70.8	73.9	85	139	41	12.1	30	yes	NO
P5	32	F	25.08	95	86.6	79	67	38	12.7	32	no	NO
P6	29	F	16.22	29.2	31.1	36	46	48	10.4	25	yes	BiPAP
P7	63	F	21.48	51.4	60.5	56	59	44	12.5	59	yes	BiPAP
**Average**	**43.6 (±12.8)**		**24.0 (±3.9)**	**60.0 (±22.6)**	**60.5 (±20.2)**	**58.6 (±19.8)**	**77.1 (±30.5)**	**42.6 (±5.2)**	**11.1 (±1.3)**	**37.8 (±11.0)**		
	**Control Subjects**											
C1	55	F	35.47	99.7	109.6	83	125	45	7.3	—	—	—
C2	49	F	24.86	133.2	119.6	118	116	41	9.1	—	—	—
C3	53	M	32.09	75	77.9	61	100	40	11.7	—	—	—
C4	29	M	26.18	141.6	106.5	156	115	39	11.1	—	—	—
C5	31	F	22.72	112.5	121	78	96	40	9.7	—	—	—
C6	20	F	23.32	117.7	121	88	102	37	10.4	—	—	—
C7	61	F	23.16	95.3	102.9	83	125	36	7.2	—	—	—
**Average**	**42.6 (±15.7)**		**26.8 (±5.0)**	**110.7 (±22.9)**	**108.4 (±15.3)**	**95.3 (±31.7)**	**111.3 (±12.0)**	**39.7 (±2.9)**	**9.5 (±1.8)**	—	—	—

**BiPAP:** bi-level positive airway pressure; **BMI:** body mass index; **CPAP:** continuous positive airway pressure; **ERT:** enzyme replacement therapy; **ETCO**_**2**_**:** end-tidal carbon dioxide; **FEV**_**1**_**:** forced expiratory volume in first second; **FVC:** forced vital capacity; **PI**_**MAX**_**:** maximal inspiratory pressure; **PE**_**MAX**_**:** maximal expiratory pressure; **V**_**E**_**:** minute ventilation.

### Dynamic MRI of the Thorax

**Figure 1:**
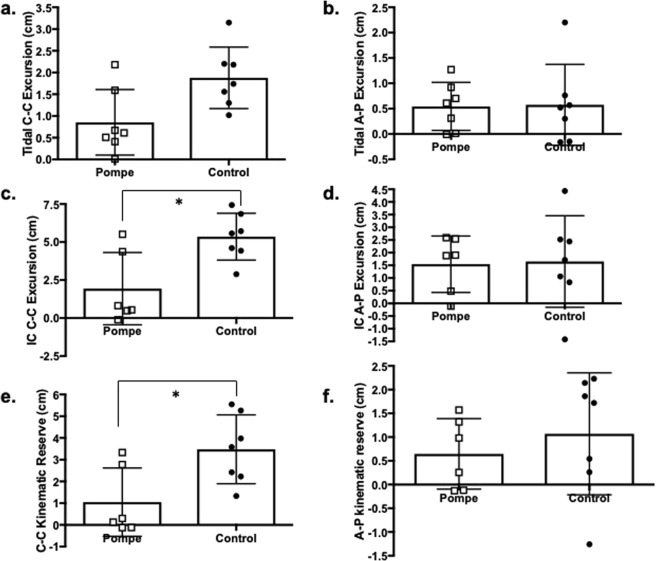
Kinematic reserve in LOPD and control subjects. Dynamic MRI was used to evaluate cranio-caudal lung height, representative of diaphragm excursion, and anterior-posterior (**A–P**) chest wall expansion. We did not find significant group differences for C -C excursion (**a**) or A-P expansion (**b**) during tidal breathing. Inspiratory capacity C-C excursion (**c**) was significantly lower for LOPD patients. In contrast, A-P expansion during inspiratory capacity (**d**) remained similar between groups. Kinematic reserve is the difference between MRI measurements of diaphragm (C-C) and chest wall (**A–P**) excursion at rest and during inspiratory capacity maneuvers. While the C-C reserve of the diaphragm (**e**) was significantly smaller for LOPD subjects, we observed a similar difference in the A-P reserve (**f**) of the chest wall muscles (*Significant group-direction interaction, F = 3.571, p < 0.05).

Table 2 summarizes the C-C and A-P distances at end-inspiration and end-expiration during tidal breathing and IC, as well as the kinematic excursion. One patient (P3) required use of BiPAP during the MRI test; therefore, IC kinematics were measured in only six LOPD subjects. There was a significant interaction between the group and direction of movement for kinematic reserve (Fig. 1e). While kinematic reserve was similar between patients and controls for A-P excursion, the C-C kinematic reserve was significantly smaller in LOPD (F = 3.571, p < 0.05).

**Table 2:** Anterior-posterior (AP) and cranio-caudal (CC) kinematic excursion measured in LOPD and CON subjects revealed that at peak inspiration, the CC height of the lung was significantly reduced in LOPD, and this reduced inspiratory height resulted in a lower CC excursion during inspiratory capacity (mean, SD).

Variable	LOPD	CON	p-value
**Tidal Breathing (n** = **7)**			
CC Inspiratory (cm)	14.2 (±3.1)	17 (±4.37)	0.111
CC Expiratory (cm)	13.4 (±3.31)	15.1 (±4.3)	0.367
CC Excursion (cm)	0.85 (±0.76)	1.88 (±0.71)	0.071
AP Inspiratory (cm)	15.22 (±2.44)	15.53 (±2.92)	0.864
AP Expiratory (cm)	14.68(±2.44)	14.96 (±2.46)	0.866
AP Excursion (cm)	0.54 (±0.47)	0.58 (±0.8)	0.897
Volume (mL)	356 (±91)	634 (±943)	0.107
**Inspiratory Capacity (n** = **6)**			
CC Inspiratory (cm)	15.6 (±3.23)	21.3 (±4.18)	0.004*
CC Expiratory (cm)	13.7 (±3.47)	15.5 (±5.5)	0.355
CC Excursion (cm)	1.93 (±2.38)	5.77 (±1.2)	0.002*
AP Inspiratory (cm)	16.36 (±2.44)	16.74 (±3.9)	0.848
AP Expiratory (cm)	14.82 (±2.86)	14.95 (±2.59)	0.945
AP Excursion (cm)	1.55 (±1.11)	1.79 (±1.94)	0.793
Volume (mL)	910 (±311)	1871 (±978)	0.053

### Single-breath ILC

**Figure 2:**
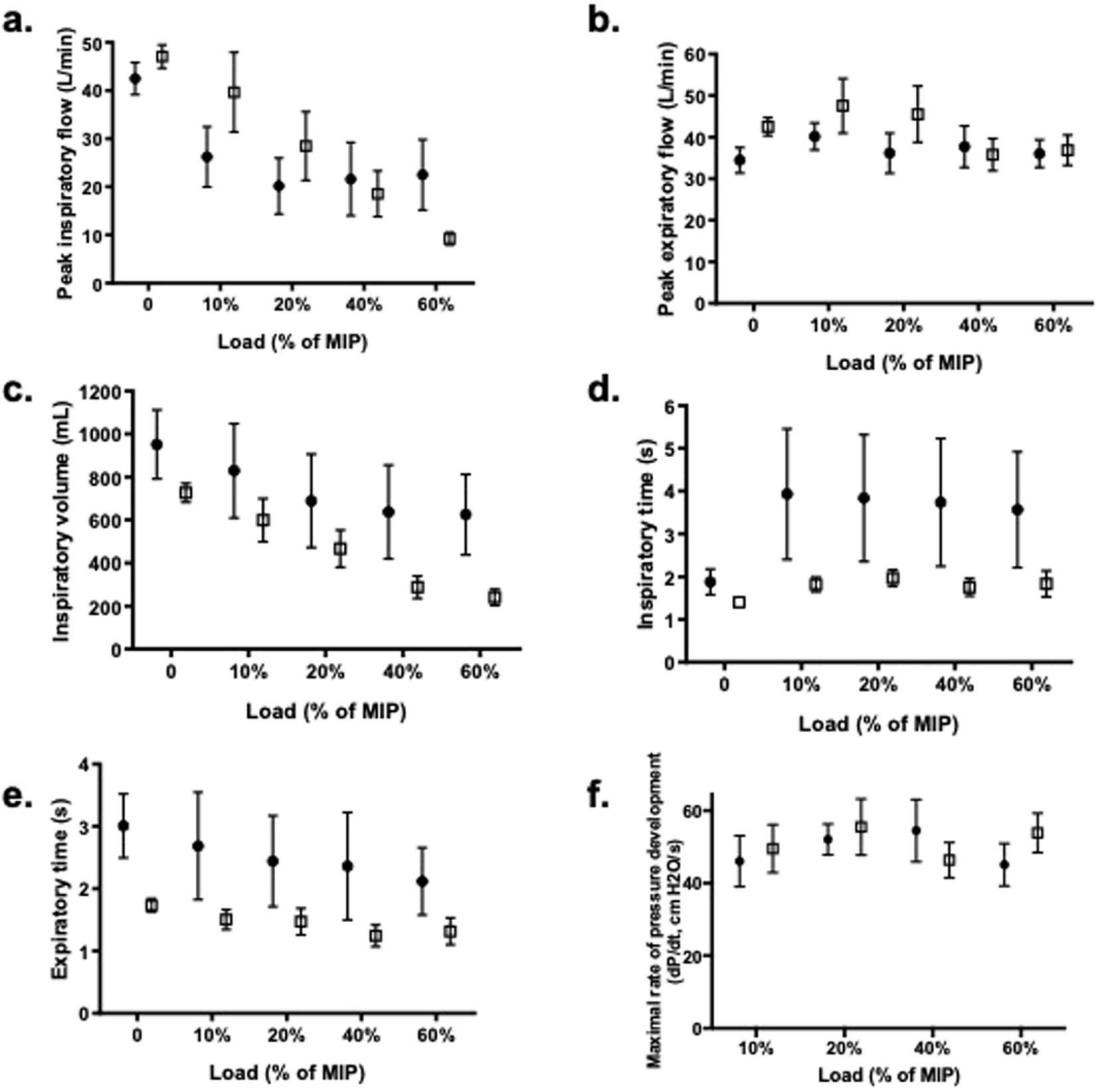
Single-breath ILC in LOPD and control subjects. Single-breath ILC responses differed according to the group (control: closed circles, Pompe: open squares) and load magnitude. During loaded breaths, peak inspiratory flow (**a**) progressively decreased in LOPD as compared to control subjects (3-way interaction, F = 2.794, p < 0.05). Compared to recovery breaths, peak expiratory flow (**b**) was lower during loaded breaths, but neither group assignment nor load magnitude affected the loaded peak expiratory flow. The inspired volume of loaded breaths (**c**) was similar for both groups at the lowest load magnitude, but in LOPD subjects, volume progressively decreased with larger load magnitudes (breath-load interaction, F = 19.403, p < 0.005). Inspiratory time (**d**) did not vary by breath type, load magnitude, or group, largely due to variability of breath timing in the control subjects. Expiratory time (**e**) was lower for load**e**d breaths, compared to unloaded controls and recovery breaths (F = 5.767, p < 0.05). Despite the group differences in flow and volume ILC, the maximal rate of inspiratory pressure development (dP/dt) of the loaded breaths (**f**), an estimate of neuromuscular activation, did not differ between the groups. (Mean, SEM depicted).

Significant single-breath ILC responses are depicted in Fig. 2. There was a significant interaction effect between the breath type, group, and load magnitude for PIF (3-way interaction, F = 2.794, p < 0.05). During loaded breaths, PIF decreased progressively in LOPD patients as the load magnitude increased, and it was significantly lower than controls at the highest load magnitude (60% of MIP, p < 0.005). In both LOPD and control subjects, PIF was highest during the recovery breaths and lowest during the loaded breaths (breath main effect, p < 0.001). No differences were found for dP/dt. On exhalation, the sample generated a significantly higher PEF during the recovery breaths, following single-breath load magnitudes>0% (breath order-load interaction, F = 6.463, p < 0.005).

There were significant breath-load interaction effects for VTI (F = 19.403, p < 0.005) and VTE (F = 7.013, p < 0.005) during single-breath ILC. Neither group preserved breath volume during the 40% and 60% load magnitudes, when compared to lower loads, unloaded control breaths, or recovery breaths (p < 0.005). Despite a lower PIF during loaded breaths, both Pompe and control subjects minimized the loss of volume by tending to lengthen TI (F = 4.186, p = 0.06). Additionally, both groups significantly shortened TE during loaded breaths (F = 5.767, p < 0.05), as compared to the unloaded control and recovery breaths.

### Endurance time

**Figure 3:**
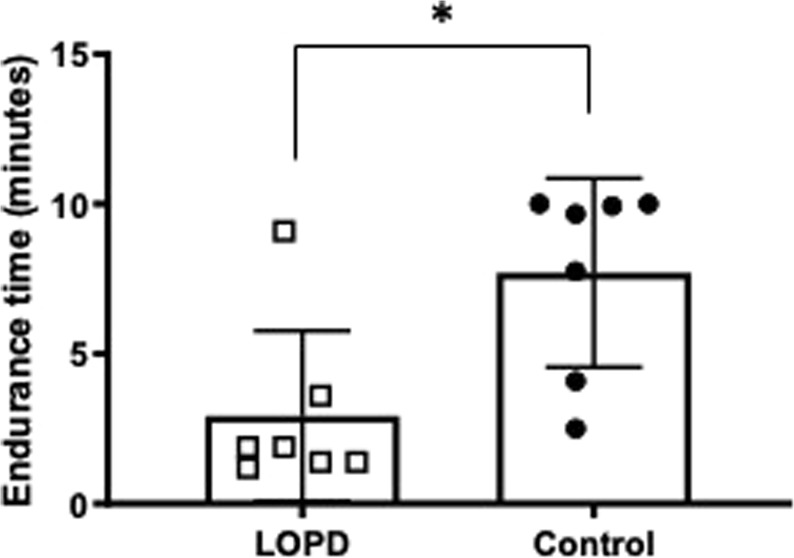
Endurance time in LOPD and control subjects. Endurance time during an inspiratory threshold load of 40% PI_MAX_ was significantly shorter in LOPD subjects (*t = 2.983, p < 0.05).

Figure 3 depicts the endurance time of the LOPD and CON subjects. Endurance tests of two control subjects (C1 and C3) were terminated early by the investigators, despite excellent inspiratory valve opening, for clinically significant elevations of blood pressure. As a group, control subjects were able to continue breathing with a 40% inspiratory load for 7.7 ± 3.1 minutes, while LOPD subjects managed the inspiratory load for 2.9 ± 2.8 minutes to failure (t = 2.983, p < 0.05).

### Associations between respiratory variables

For the entire sample, PI_MAX_ and FVC correlated significantly (r = 0.858, p < 0.001), along with maximal diaphragm descent and Tlim (r = 0.827, p < 0.005). Inspiratory volume ILC significantly correlated to flow (r = 0.907, p < 0.005), as well as inspiratory and expiratory timing (volume-TI: r = 0.846, p < 0.001 volume-TE: r = 0.852, p < 0.001). In LOPD, volume and inspiratory flow ILC correlated with maximal chest wall expansion (volume-AP-excursion: r = 0.943, p < 0.005, inspiratory flow-AP-excursion: r = 0.962, p < 0.005) but not with diaphragm descent (volume-CC-excursion: r = 0.122, p = 0.82, inspiratory flow-CC-excursion: r = 0.312, p = 0.55).

## Discussion

The key findings of these research tests of dynamic inspiratory muscle function indicated kinematic evidence of diaphragm paresis, accompanied by altered inspiratory endurance and strength in patients with LOPD. The ILC tests indicate an impaired ability for LOPD patients to generate inspiratory flow with an unanticipated inspiratory load. However, a compensatory prolongation of TI helped to preserve volume during the loaded breath. We also observed flow and volume ILC were associated with MRI tests of maximal chest wall expansion, but not with diaphragm kinematics, of patients. While likely more useful as research tools than in daily clinical practice, these tests appear to capture distinct aspects of dynamic respiratory muscle function beyond standard pulmonary function tests, which could help clarify how patients maintain minute ventilation despite gradual LOPD disease progression.

We used dynamic MRI to evaluate kinematics of breathing at rest and during inspiratory capacity. The C-C kinematic reserve represented the difference between resting diaphragm excursion and maximal diaphragm excursion, and it indicated the presence of diaphragmatic paresis in LOPD. On the other hand, A-P resting and reserve excursion did not differ significantly between patients and controls, indicating relative preservation of chest wall expansion in patients.

Existing studies in adult onset Pompe also contrasted MRI to conventional lung function tests^3,7^, but many prior efforts focused on static breath-holds. Our findings of early diaphragmatic paresis in LOPD were also in agreement with existing respiratory studies using opto-electronic plethysmography (OEP)^21^ and static MRI^3,6^. Additional advantages to using dynamic MR modes include the ability to average the kinematics over multiple breaths in a short period of time and the removal of possible confounding effects of elastic recoil on breath-holding^22^. MRI may be suitable for multi-site research studies that cannot accommodate the substantial equipment costs and data analyses required with OEP. For example, children with infantile-onset Pompe disease tolerated dynamic respiratory MRI in a clinical trial of AAV1 gene therapy^11^, while on ventilatory support and for brief periods of CPAP or independent breathing.

Thoracic MRI may also offer scientists insights regarding the presence of fat or fibrotic incursions in the respiratory muscles^23,24^. However, there are limitations to MRI, particularly in the context of clinical care. In the current study, one LOPD subject with inspiratory muscle weakness and absent C-C excursion required the use of BiPAP to image tidal breathing. Therefore, while ultrasonography can yield lower tissue resolution and an insufficient window in some patients, it may be preferred in clinical settings or to monitor moderate to severe diaphragmatic involvement^25,26^. Additionally, sniff nasal inspiratory pressure (SNIP) reflects pressure associated with a maximal effort sniff. While the current study focused on MIP due to its common inclusion as a clinical study endpoint, the SNIP test is well tolerated in neuromuscular diseases^27^ with potential predictive value for respiratory morbidity^28^.

Tlim was restricted in LOPD and correlated strongly with conventional lung function tests as well as maximum diaphragm excursion during MRI. In contrast to the MRI tests, all LOPD subjects were able to complete Tlim. Advantages of endurance tests are their ability to discern changes in pressure generation that do not involve a static hold and become less effort-dependent with approaching task failure^29^. Since the respiratory pattern and mouth pressure were standardized, the endurance test has been considered less susceptible to learning effects, in healthy subjects and patients with neuromuscular disease^30,31^. As loads increase in magnitude or are sustained to fatigue, inspiratory flow and inspiratory time progressively diminish^32^. Subjects reached task failure (Tlim) when they could not generate sufficient pressure to continue to open the inspiratory valve and receive airflow^32^. Longitudinal changes in Tlim may indicate an external factor (e.g. disease, training, medication) altered the endurance Tlim, and further study is needed on whether tests of threshold loading can longitudinally track changes respiratory muscle function with either progressive disease or in response to therapeutic interventions such as inspiratory muscle training. Indeed, recent reports suggest inspiratory muscle training can lead to strength gains, in some individuals with LOPD^33^, We previously observed that difficult to wean ICU patients who failed to wean after inspiratory muscle training had lower flow/volume ILC that failed to improve with training^34^. We speculate that Tlim and/or ILC tests could help scientists further identify inspiratory flow and volume signatures associated with an incomplete strengthening or functional benefit from inspiratory muscle training^16,31^.

Compensatory responses to a single-breath threshold load include reductions in inspiratory flow and volume, with prolongation of TI and reduction of TE^15,35^. We observed these modifications during single-breath ILC, with some distinctions in the LOPD group. Consistent with other neuromuscular diseases^36^, both TI and TE were slightly shorter in LOPD subjects than CON. As the magnitude of a single breath load increased, both PIF and volume dropped significantly in LOPD subjects. With inspiratory challenges, the diaphragm responds by increasing the firing frequency of active motor units and by recruiting previously inactive diaphragm motor units^37^. Additionally, accessory muscle motor units that are largely inactive at rest are recruited^38,39^. In healthy individuals, recruitment of chest wall muscles is thought to offset diaphragm fatigue during sustained inspiratory loading^40^. In LOPD, we noted a strong association between increased chest wall kinematic motion and the PIF and VTI generated during single-breath ILCs. We speculate that chest wall respiratory recruitment may influence single-breath ILC responses in LOPD, because early, severe diaphragm paresis minimizes the ability of the diaphragm to compensate to the transient, unexpected load.

Fifty-seven percent of the LOPD subjects required nighttime non-invasive respiratory support, which itself indicates a loss of respiratory neuromuscular reserve^41^. Both neurological and muscular contributions to respiratory dysfunction in LOPD have been reported in the literature. Diaphragm dysfunction is a distinguishing characteristic of LOPD identified even among mildly affected LOPD patients^42^, but an enhanced inspiratory neural drive may be able to preserve independent breathing in early diaphragm dysfunction^43^. Neuromuscular activity, as estimated by dP/dt, was similar for Pompe and control subjects during ILC^16,44^. ILC loading was administered as a proportion of MIP, rather than a fixed value, indicating that relative proportional loads elicited a similar proportional neuromuscular activation. While the small sample did not permit a more rigorous comparison of neuromuscular activation in the patients who used nighttime support^43,45–48^, the use of nighttime ventilation in LOPD improves both ventilation and oxygenation during sleep, without an appreciable change in respiratory muscle strength^49^. In the current study, the similar dP/dt and prolongation of TI with threshold loading in both LOPD and control subjects indicates that both groups used a similar motor control strategy to overcome inspiratory loads, and we speculate this could indicate a loss of respiratory motor units in Pompe, as opposed to an altered functioning of the motor neurons^43,45–48^.

We acknowledge some limitations to this study. FVC and respiratory pressures were not tested in supine. Subject fatigue was the primary consideration for this decision, and IC in the MRI was computed instead. While the requirement to maintain a consistent respiratory rate and duty cycle during the endurance test has been criticized by some, it reduces variability in the breathing pattern, namely an early prolongation of exhalation, which could artificially prolong Tlim^50^. It could be argued that both the Tlim test could be considered as effort-dependent as lung function and maximal respiratory pressure tests^51^. A distinguishing factor is the binary nature of the threshold valve, which blocks inspiratory flow for inspiratory pressures below the threshold. Additionally, the threshold valve mechanics and limited compensatory strategies for clinical populations reduce effort and learning effects on ILC and Tlim. Finally, the finite time and energy of LOPD patients required us to limit the range of respiratory tests. Specifically, invasive tests of evoked diaphragmatic pressure would provide additional mechanistic insights on non-voluntary activation of the diaphragm, while SNIP and diaphragm ultrasound tests offer clinical insights into dynamic diaphragm function.

In summary, our findings provide evidence of early dynamic diaphragmatic dysfunction in LOPD, compared with controls. This provides critical evidence that diaphragm dysfunction in Pompe alters not only static force generation, but also impairs the ability to preserve minute ventilation during transient or sustained inspiratory loads. In turn, a lowered dynamic inspiratory muscle reserve may ultimately affect a patient’s ability to resist physiological stressors such as bronchoconstriction or a narrowed airway during sleep, placing them at higher risk for failure. Future studies should distinguish how dynamic respiratory muscle function changes with disease progression.

## Methods

### Subjects

Fourteen subjects volunteered to participate. Seven subjects had a diagnosis of LOPD confirmed by genetic testing or enzyme activity assays (age 29–63 years, 5 women), and the remaining seven were age- and gender-matched unaffected control subjects (age 20–61 years). Based upon a power analysis of preliminary data on the correlation between PI_MAX_ and the volume ILC, at least 6 subjects per group would be needed for α = 0.05, (1-β) = 0.9, (ρ = 0.68). The study design and procedures were approved by the University of Florida Institutional Review Board and conducted in accordance with principles established in the 1964 Declaration of Helsinki and its subsequent amendments. All participants provided their informed consent prior to inclusion in the study. The study was registered in February 2015 (NCT02354664).

Exclusionary characteristics for patient participation included: pre-existing obstructive lung disease or asthma, a forced vital capacity (FVC) < 30% of age/gender predicted values, requirement for mechanical ventilation while awake and upright, inability to travel to the study site, pregnancy, or any other chronic medical condition that would interfere with study participation.

### Experimental protocol

Each subject participated in a single 6-hour study visit, divided into two days. The following tests were included: (1) Clinical pulmonary function and maximal respiratory pressure tests, (2) Dynamic MRI of the thorax, (3) Resting breathing pattern, (4) ILC to unexpected, single-breath loads, (5) ILC to sustained loads, and (6) inspiratory endurance.

#### Clinical pulmonary function and maximal respiratory pressure tests

Upright forced vital capacity (FVC) was completed per American Thoracic Society guidelines^52^ and compared to references for age, gender and ethnicity^53^. Tests were repeated until three technically-acceptable efforts were acquired within 5% variability. In addition, subjects performed PI_MAX_ maneuvers from residual volume and maximal expiratory pressure (PE_MAX_) from total lung capacity, per American Thoracic Society testing guidelines^52^. Each trial was repeated after a>30 second break, until <10% variability was achieved between three trials (typically achieved within 3–7 trials).

#### Dynamic MRI of the thorax

Patients were positioned supine in a 3.0T research MR scanner (Philips Achieva) using a 32-channel cardiac coil. We customized a dynamic MR sequence, based on techniques validated in pulmonary disease^54,55^. Triplanar localizer images were acquired during a maximal inspiratory breath-hold, to establish the optimum field of view for the dynamic scans. A T2-weighted, balanced, gradient echo sequence with short repetition time and retrospective gating was employed to obtain fast imagery with excellent tissue resolution^55,56^, (TR = 2.97 ms, TE = 1.62ms, slice thickness: 8mm, ~5 frames/sec). Separate dynamic scans captured resting tidal breathing and inspiratory capacity maneuvers, in the coronal and right sagittal planes. Each tidal breathing acquisition lasted one minute and captured 12–15 tidal breaths.

During each MRI scan, respiratory parameters were collected simultaneously, using a flowmeter (Capnostat, Philips Respironics) connected to the patient via facemask and placed in series with a respiratory monitor (Nico, Philips Respironics) and laptop computer. Respiratory data acquisition occurred at 100 Hz frequency. Extended tubing was required to maintain the data acquisition equipment behind the 5 Gauss line, which added 24 mL of dead space to the respiratory circuit.

#### Resting breathing pattern

Tidal breathing pattern was assessed with subjects seated wearing a sealed facemask and in a relaxed state listening to music. Approximately 10 minutes of steady-state, resting data was sampled at 100 Hz with a respiratory monitor (CO2SMO, Philips Respironics) attached to a laptop. The resting respiratory rate was used as the breath rate for the endurance test.

#### Single-breath ILC

Single-breath ILC was tested using a custom apparatus that contained 4 pressure-threshold devices (Threshold IMT, Philips-Respironics) connected to the patient circuit, which consisted of a pneumotachograph (HR1000, Hans Rudolph) with phalange mouthpiece and nose clip. Some of the pressure-threshold devices contained a modified spring to provide higher threshold loads (14–82 cm H_2_O). Inspiratory loads of either 10, 20, 40, or 60% of PI_MAX_ (or a no-load control) were applied during exhalation, for a single inspiratory effort. Subjects were separated from the testing apparatus by a screen. Six presentations of each load were applied in random order, and a minimum of 4 and maximum of 12 unloaded breaths separated each ILC breath.

#### Respiratory muscle endurance test

Endurance was evaluated by identifying the time limit (Tlim) that a participant could maintain breathing with a submaximal inspiratory threshold load, as described previously in Duchenne muscular dystrophy^31^. Subjects rested a minimum of 30 minutes prior to the test. Subjects used a mouthpiece and nose clip connected to pneumotachograph and pressure transducer. Respiratory parameters and breathing kinematics were sampled at 100 Hz (PowerLab S35/16, AD Instruments). A pressure-threshold inspiratory load equivalent to 40% of PI_MAX_ was placed on the inspiratory port of the mouthpiece. The respiratory rate was set to each subject’s self-selected resting breath rate with a 50% duty cycle, and a two-tone metronome timed inhalation and exhalation. Oxygen saturation, end-tidal CO_2_ (ETCO_2_), heart rate, tidal volume, and respiratory rate were continuously monitored, with blood pressure readings every 2 minutes. Subjects received encouragement to maintain the target rate and mouth pressure. The test ended when one of the following conditions was met: (1) the inspiratory port did not open for 3 continuous breaths, (2) significant changes in heart rate or blood pressure outside of conventional exercise testing limits, (3) the subject reported intolerance or inability to continue, or (4) the subject reached 10 minutes of continuous loaded breathing.

### Data analysis

#### Dynamic MRI of the thorax

**Figure 4:**
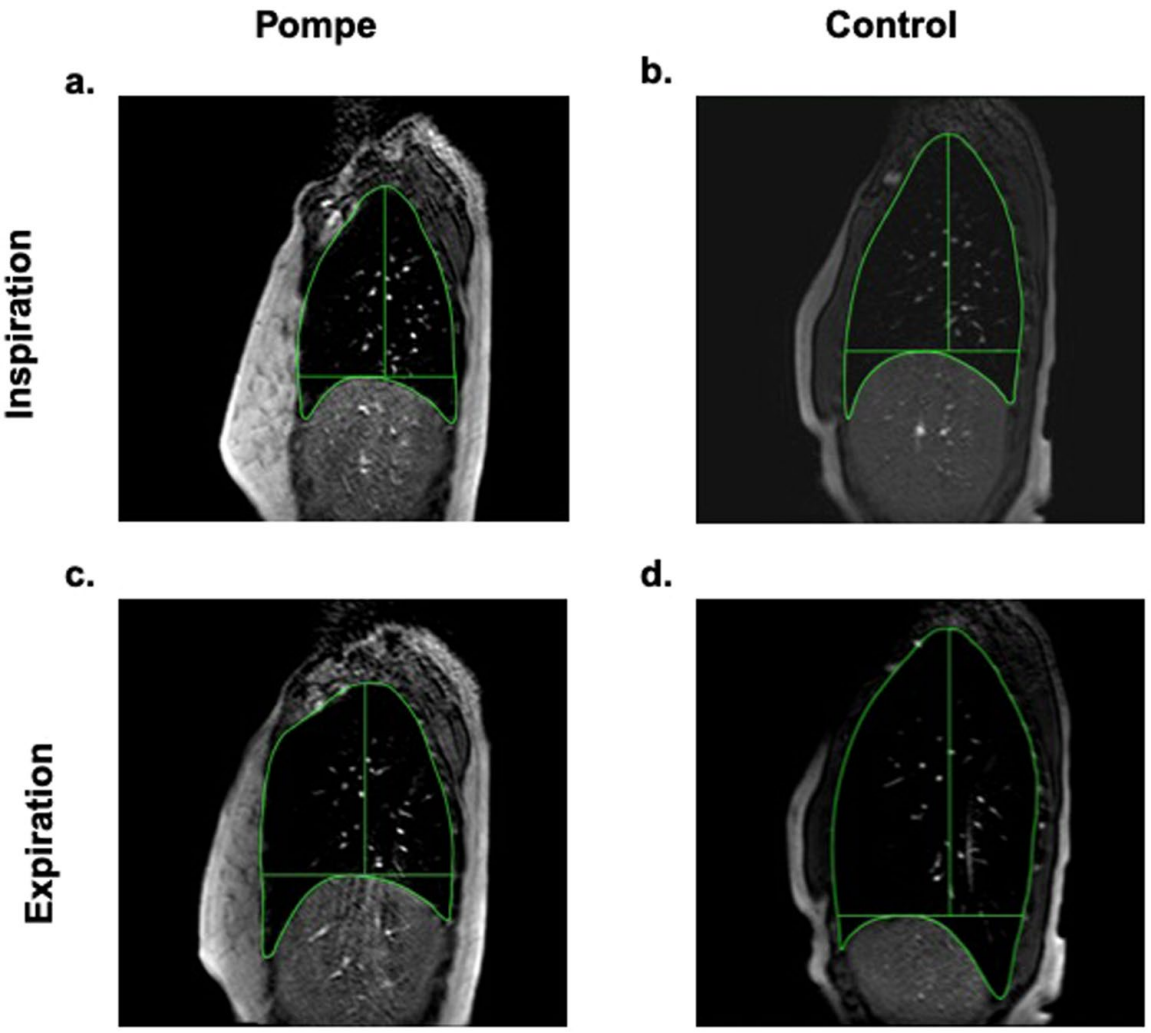
Right sagittal thoracic MRI images at end-inspiration and end-expiration during an inspiratory capacity maneuver. At end-exhalation, lung volumes were similar in (**a**) subjects with late-onset Pompe disease (subject 3 shown) and in (**b**) healthy controls (subject 14 shown). (**c**) In Pompe disease, maximal inspiratory volume generation occurred exclusively through anterior-posterior (A-P) expansion of the chest wall. In contrast, (**d**) control subject generated volume primarily through diaphragm descent, measured as increased cranio-caudal (C-C) distance.

MRI images were uploaded to a secure database and then analyzed offline with an open-source DICOM viewer (Osirix, Pixmeo) by a trained assistant blinded to subject group. We evaluated the right sagittal plane, since height of the diaphragm differs by ventral-dorsal position (See Fig. 4)^57^. The first ten uninterrupted breaths in each breathing sequence were evaluated. The cranio-caudal length (C-C) and anterior-posterior width (A-P) of the thorax were measured in each breath at end-inhalation and end-exhalation and represented the positions of the diaphragm and chest wall, respectively. Kinematic excursion was calculated as the difference between inspiration and expiration distance. Figure 4 illustrates sagittal plane images in representative unaffected and LOPD subjects, during tidal breathing and inspiratory capacity (IC) maneuvers. MRI images were time-matched to the respiratory measures. The respiratory measurements were averaged for tidal breathing and for 3 IC maneuvers.

#### Single-breath ILC

At each load magnitude, the breaths with the highest and lowest volume were excluded. The remaining 4 presentations were averaged for peak inspiratory and expiratory flow (PIF, PEF), inhaled and exhaled volume (VTI, VTE), inspiratory and expiratory times (TI, TE), and maximal date of inspiratory pressure development (dP/dt). The dP/dt was the positive peak of the pressure-time derivative^44^. In addition, we averaged the breaths before (unloaded control) and after (recovery) the loaded breath.

### Statistical analysis

Analyses were performed with the IBM SPSS Statistics 21.0 statistical package (IBM Corp., Armonk, NY, USA). Group demographics were compared using 2-sample t-tests. For MRI studies, we used 3-way repeated measures ANOVAs to evaluate the kinematic excursion of the respiratory muscles (2 subject groups, 2 directions of movement, 2 conditions -rest vs IC), and 2-way ANOVA to compare the volumetrics (2 groups, 2 respiratory conditions). Single-breath ILC was assessed using a 3-way ANOVA (2 groups, 3 breath types, 5 load magnitudes). A 2-way ANOVA tested dP/dt of the loaded breaths. With ANOVA testing, we corrected violations of sphericity with Greenhouse-Geiser adjustments. Tukey’s HSD post hoc test was used to assess individual ANOVA differences. Two-sample t-tests were used to compare the Tlim of the Pompe subjects and controls. Pearson’s correlations were used to evaluate the linear relationships between the pulmonary function, imaging, ILC, and endurance variables. The alpha level was 0.05, with the exception of correlations, which used a more stringent threshold of p < 0.005 to correct for multiple correlations.

## Data Availability

The datasets generated during the current study are available from the corresponding author on reasonable request.
